# Pancreatic Beta Cell Function in Infants Varies by Maternal Weight

**DOI:** 10.3390/metabo14040208

**Published:** 2024-04-06

**Authors:** Lisa R. Staimez, Anubrati Dutta, Yara S. Beyh, Ruby Gupta, Hari Krishna Noule, Vyakaranam Sapna, Kothapally Deepa, Aryeh D. Stein, K.M. Venkat Narayan, Dorairaj Prabhakaran, Basany Kalpana, Poornima Prabhakaran

**Affiliations:** 1 Emory Global Diabetes Research Center, Woodruff Health Sciences Center, Emory University, Atlanta, GA 30322, USA; yara.beyh@emory.edu (Y.S.B.);; 2 Hubert Department of Global Health, Rollins School of Public Health, Emory University, 1518 Clifton Rd NE, Atlanta, GA 30322, USA; 3 Centre for Chronic Disease Control, New Delhi 110016, Delhi, India; anubrati@ccdcindia.org (A.D.); ruby@ccdcindia.org (R.G.); dprabhakaran@ccdcindia.org (D.P.); poornima.prabhakaran@ccdcindia.org (P.P.); 4 Share India, MediCiti Institute for Medical Sciences, Medchal 501401, Telangana, India; sapna_biochm@mims.edu.in (V.S.); hod.kalpana_gyanec@mims.edu.in (B.K.); 5 Administrative Staff College of India, Hyderabad 500034, Telanaga, India; 6 Public Health Foundation of India, Gurgaon 110030, Haryana, India

**Keywords:** pancreatic beta cell function, infancy, developmental origins, pregnancy, weight

## Abstract

The Asian Indian Beta Cell function (ABCs) in Infants Study examined the associations of maternal weight on infant pancreatic beta cell function across 7 months postpartum. Pregnant women aged 18–35 years were recruited in Hyderabad, India. Women were classified by first trimester weight as underweight (UW), BMI < 18.5 kg/m^2^; normal weight (NW), BMI 18.5–22.9 kg/m^2^; or overweight (OW), BMI 23.0 through <28.5 kg/m^2^. At age > 7 months, infants had an oral glucose tolerance test (OGTT, 1.75 g glucose/kg bodyweight) following a 3 h fast. Infant blood samples were assayed for C-peptide and glucose. Infant beta cell function (HOMA2-B; disposition index, DI) and insulin resistance (HOMA2-IR) were compared across maternal weight groups. Mothers (UW *n* = 63; NW *n* = 43; OW *n* = 29) had similar age at delivery and second trimester 50 g glucose challenge test results. Cord HOMA2-B values were 51% greater for I_UW_ (83.5, SD 55.2) and 44% greater for I_OW_ (79.9, SD 60.8) vs. I_NW_ (55.4, SD 51.5), forming a U-shaped relationship between maternal weight and HOMA2-B. No qualitative differences in HOMA2-IR were found at birth. However, at 7 months postpartum, HOMA2-IR changed most within I_UW_ (−64% median reduction) and changed the least in I_OW_ (−7% median reduction). At seven months postpartum, DI was higher in I_UW_ vs. the other groups (geometric mean I_UW_ 1.9 SD 2.5; I_NW_ 1.3 SD 2.6 or vs. I_OW_ mean 1.2 SD 3.7), reflecting a +49% difference in DI. Evidence from this study illustrates adaptations in the pancreatic functional response of infants associated with the maternal nutritional environment.

## 1. Introduction

The increase in type 2 diabetes globally is accompanied by diabetes-related complications and losses in quality of life [1,2,3]. Type 2 diabetes develops when pancreatic endocrine cells can no longer respond adequately to metabolic needs and maintain normal glucose levels. Pancreatic beta cells must produce adequate amounts of insulin, even across extended time periods of high metabolic demand and stress, including elevated insulin resistance. The factors leading to beta cell growth and failure are potential targets for diabetes prevention and management.

Intrauterine factors can support or inhibit health in the next generation. Models of developmental origins in health and disease (DOHaD) emphasize that conditions in pregnancy can influence the development of physiological mechanisms driving metabolic response in adulthood. The nutritional conditions of pregnancy, both under- and over-nutrition during pregnancy, may impact the risk of diabetes later in life [4,5,6]. A maternal environment of undernutrition during fetal development and infancy may alter the metabolism of infants and their risk for chronic disease later in life through a number of cellular modifications in key organs during critical developmental periods [7,8]. Proposed cellular modifications include epigenetic changes, i.e., changes to DNA methylation, histone modifications, and/or noncoding RNA expression or activity, that can impact the gene expression of genes controlling metabolism [9,10,11,12]. These cellular modifications have been proposed across several tissues involved with diabetes development, including pancreas, adipose, and liver. Maternal obesity may affect the maternal circulating levels of adipokines produced from adipose tissue. Animal models have demonstrated that changes in the production of leptin and adiponectin impact the development of hypothalamic neurocircuits, resulting in permanent changes around preferences for extra-palatable foods and lower energy expenditure levels [13]. Similarly, maternal hyperglycemia in overnutrition increases infant risk of altered metabolic features, including increased intra-abdominal fat, with subsequent fibrosis and hypertrophy of fetal islet tissue and insulin hypersecretion [14,15].

We examined the role of maternal underweight and overweight on infant beta cell function in the first year of life. We collected longitudinal data from South Asian pregnant women and their infants to examine the role of maternal weight class and pregnancy weight gain on infant insulin secretion during the first year of life. South Asians are at high risk for diabetes compared to other ethnicities, even at comparable BMI levels or younger ages [16,17]. The distribution of maternal weight among South Asians includes a higher proportion of underweight women compared to populations of white European descent. We hypothesized that marked differences would be observed in the beta cell function of infants from underweight mothers (I_UW_) compared to infants from normal weight (I_NW_) or from overweight mothers (I_OW_) in the first year of life.

## 2. Materials and Methods

Study Population. Women aged 18–35 years were recruited from an obstetric clinic in Hyderabad, India, into the Asian Indian Beta Cells (ABCs) in Infants Study. As an ancillary study to an existing pregnancy cohort study [18], the ABCs in Infants Study was designed to follow a subset of women from a pregnancy cohort, the HEaLthy Pregnancy Outcome Study (HELP Study), at Mediciti Institute of Medical Sciences, a rural tertiary care teaching hospital, Ghanpur, Hyderabad, Telangana State, India.

**Figure 1:**
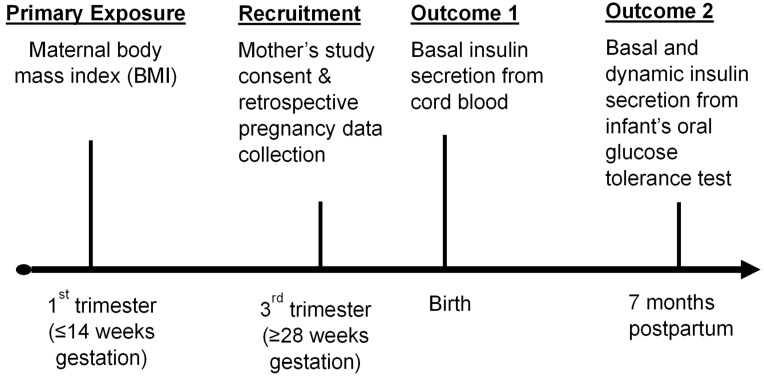
Design and timeline for the Asian Indian Beta Cells (ABCs) in Infants Study.

Women who provided consent to participate in this optional, add-on study were informed of key study procedures, including cord blood sampling at the time of delivery. Women were asked for reconsent of their and their infant’s study participation prior to the second study visit, which included infant blood sampling at 7 months postpartum as part of an infant oral glucose tolerance test (Figure 1). Data from each mother–child dyad spanned from first trimester of pregnancy to 8 months postpartum. The study was approved by the Institutional Review Board (IRB) of Emory University, Atlanta, GA, USA, the independent ethics committee (IEC) of the Centre for Chronic Disease Control (CCDC, Delhi, India), and the IEC of the Society for Health Allied Research and Education-India (Share India), Mediciti Institute of Medical Sciences, Hyderabad, India.

Inclusion Criteria. Women in their third trimester who had clinic records of the first trimester (≤13 weeks) BMI were included. Women who were underweight were oversampled to 50% of the recruited study sample. Weight groups were defined according to Asian-specific cut points: underweight (UW) BMI < 18.5 kg/m^2^; normal weight (NW) BMI 18.5 through <23.0 kg/m^2^; and overweight (OW) BMI 23.0 through <27.0 kg/m^2^ [19]. We further included two women who had a BMI of less than 28.5 kg/m2, and thus, the overweight category ranged from 23.0 through <28.5 kg/m^2^. 

Exclusion Criteria. Mothers were excluded from the study for multiple pregnancies; for having maternal age < 18 years or >35 years; or for pre-existing conditions including the following: any known history of bleeding tendency or thrombosis; high-risk pregnancies or any known bleeding episode in the current pregnancy; hypertension; diagnosed heart disease; cancer; medical condition(s) which could affect the safety of mother/infant; or any known allergies to the test materials used in the study. 

Study Procedures. Women in their third trimester were invited to enroll in the study. Among those who provided consent, cord blood samples were collected after delivery and before the expulsion of the placenta in both vaginal and caesarean deliveries. Umbilical venous blood was collected from the double-clamped cord immediately after delivery of the placenta. Samples in fluoride-treated tubes were immediately taken to the hospital lab, where the glucose level was assessed using a One Touch Verio glucometer (LifeScan, Malvern, PA, USA). Blood samples were not included in data analysis if there was a 20 min or greater delay in cord blood collection or if antenatal glucocorticoids had been administered in the 24 h before birth.

Woman–infant dyads were followed during clinic visits, and at 7 months postpartum, the women were invited to participate in that portion of the study. Mothers who provided consent were provided instructions on how to best prepare the infant for a study visit that included an infant OGTT [20]. Infants were administered a standardized 30 min oral glucose tolerance test [1.75 g glucose/kg body weight (33% *w/v*, i.e., concentration of glucose as weight glucose for volume water)] to assess early-phase insulin secretion following a 3 h fast. Secretion was measured by assessing the change in insulin (or C-peptide levels relative to the change in glucose levels across the first 30 min of physiologic response to the glucose bolus (see Measures section below). Infants were allowed 10 min to ingest the glucose. All infants completed their glucose drink within the allotted time. Venous blood samples were collected at 0 and 30 min of OGTT by a skilled pediatric phlebotomist. Nearly all mother–infant dyads remained in the study after birth and provided OGTT sampling at 7 months postpartum (i.e., 92% retention). Blood samples were analyzed for glucose on site. On the day of the OGTT, women were asked about their child’s diet and medication use for the past 2 days. After the enrollment of 155 women in pregnancy, the final cohort of mother–infant dyads for analysis consisted of 135 dyads (see Section 3 for more information).

Laboratory Procedures. Insulin and C-peptide were assayed in cord and infant blood samples by ELISA, using solid-phase two-site enzyme immunoassay based on the direct sandwich technique. Kits and controls were procured from Mercodia AB, Uppasala, Sweden. Glucose was measured by glucometer (electroenzymatic with glucose oxidase). The laboratory (Centre for Chronic Disease Control, India) participates in the UK National External Quality Assurance Scheme for insulin. Two level of controls were run with every batch of samples analyzed. The intra-assay coefficient of variation for insulin was 5.55% and 2.28% and the inter-assay coefficient of variation was 11.1% and 10.1% at mean values of 8.02 and 58.35 mU/L, respectively. The intra-assay coefficient of variation for C-peptide was 3.76% and 4.43% and the inter-assay coefficient of variation was 10.7% and 7.0% at mean values of 440 and 1400 pmol/L, respectively. Fasting and postprandial samples were analyzed in the same plate to control for inter-plate variation. 

Measures. Primary Exposures: Two measures for maternal nutritional status were used: (1) first trimester BMI and (2) pregnancy weight gain, analyzed both continuously and categorically. First trimester BMI was based on weight and height obtained from medical records previously collected by trained workers for the parent HELP Study. Gestational weight gain was calculated from maternal weight measured at delivery and the earliest first trimester pregnancy weight. Adequate weight gain was defined as that at or above the Institute of Medicine recommended threshold [21]. 

Primary Outcome. Beta cell function in infants was the primary outcome, defined as the amount of insulin secreted relative to prevailing levels of insulin sensitivity. Dynamic beta cell function was calculated according to several indices utilizing measures from 0 and 30 min time points of the OGTT: the insulinogenic index [ΔC-pep_0–30_/ΔGlu_0–30_]; and the oral disposition index ([ΔC-pep_0–30_/ΔGlu_0–30_] × [1/Ins_0_]. Basal beta cell function was assessed by HOMA2-B [22], calculated from cord blood at the time of birth as well as from infant blood 7 months postpartum. Insulin resistance and insulin sensitivity were measured by (1) the homeostasis model assessment for insulin resistance (HOMA2-IR) [22] and (2) 1/fasting insulin (1/Ins_0_).

Covariates. As part of the HELP study, all women had a first trimester random blood glucose measurement and a second trimester 50 g glucose challenge test. Other measures from pregnancy included family history of diabetes across first degree relatives; and demographic factors including maternal age, income, and education. The date of the last menstrual period (LMP) was used to estimate the date of conception. Infant birth measures included gestational age (GA), based on the date of LMP and prospective observation of the date of delivery. Preterm birth was defined as gestational age < 37 weeks. Birth weight for age, length for age, and head circumference for age Z scores were computed [Z = (x−mean)/standard deviation, SD], using WHO Child Growth Standards [23]. BMI was calculated as weight/height^2^ (kg/m^2^). 

### Analysis

We planned to invite and enroll at least 150 women into the study, expecting a 20% loss to follow-up (e.g., no response, withdrawal from study, etc.), and we enrolled 155 women. Expected differences in infant beta cell function between underweight mothers and normal weight mothers were based on mean estimates of pancreatic insulin response in children across different nutritional status categories in childhood [24]. Using the reported standard deviation of the insulinogenic index (∆insulin_0–30min_/∆glucose_0–30min_ from the OGTT) for children who were small for gestational age (SGA, 0.28 ± 0.15) vs. average (0.40 ± 0.24), we calculated that a sample of 60 infants from underweight mothers compared to a sample of 30 infants from normal weight mothers would achieve over 80% power to detect a 0.13 effect size at a significance level (alpha) of 0.050 using a two-sided two-sample unequal-variance *t*-test. Estimates of the insulinogenic index in large for gestational age (LGA) infants were not available, so we assumed a similar standard deviation in the insulinogenic for LGA as infants of average size, and a sample comparing 30 infants from normal weight mothers to 30 infants from overweight mothers would achieve over 80% power to detect a 0.15 effect size at a significance level (alpha) of 0.050 using a two-sided two-sample unequal-variance *t*-test. For this study, we substituted C-peptide for the calculation of the insulinogenic index (∆C-peptide_0–30min_/∆glucose_0–30min_ from the OGTT), an approach with lower variance around means compared to index calculations using insulin [25].

Analyses were conducted in SAS 9.4, and data were expressed as mean and SD. Based on recommended methods by statistical experts, which include the reduced emphasis of *p*-values to determine study results, [26,27,28] mothers’ characteristics and infants’ characteristics were compared across BMI groups based on percent differences of means between groups (i.e., underweight vs. normal weight; and overweight vs. normal weight) and based on differences in frequencies or Z scores between groups. Differences of 15% or larger were considered as between-group differences and further evaluated relative to measures of precision. In addition, we assessed longitudinal, within-person change (i.e., change in HOMA2-B from birth to 7 months of age) as percent change for each infant. The mean and median percent change were then calculated for each BMI group. Continuous variables that were not normally distributed were log transformed to meet the assumptions of tests used. For measures of beta cell function, the hyperbolic relationship of insulin secretion and insulin sensitivity [29] was assessed using linear regression to estimate ln(∆I_0_–I_30_/∆G_0_–G_30_) as a function ln(1/fasting insulin) or ln (1/HOMA2-IR). A hyperbolic relationship was confirmed if the slope was approximately equal to −1 and 95% CI excluded 0 [30,31]. Multivariate linear regression was used to examine the exposure of maternal weight and the dependent variable of beta cell function (i.e., HOMA2-B, the insulinogenic index, and the oral disposition index) and the dependent variable of insulin resistance (i.e., HOMA2-IR). 

## 3. Results

Among 155 women who consented to participate in the study during pregnancy, 135 completed follow-up with their infant (92% retention). Average follow-up time was 7 months postpartum (mean 7.4 months SD 0.9). The remaining women–infant dyads were lost to follow-up (n = 19) or did not have blood samples suitable for analysis (lysed blood sample, n = 1) and were excluded from the analytic study sample. Loss to follow-up was similar between underweight (n = 10, 16%) and normal weight (n = 8, 19%) groups; it was lower in the overweight group (n = 2, 7%). The final cohort of mother–infant dyads who had baseline and follow-up measures included underweight women (n = 63), normal weight women (n = 43), and overweight women (n = 29).

The mean age of all women at the time of delivery was 23.1 years (SD 2.9), similar across weight classes (UW: 23.0 years SE 0.4; NW: 22.7 years SE 0.4 OW: 23.8 SE 0.6). The mean weight by trimester remained ordered from UW (least) to OW (greatest) across pregnancy trimesters, although differences narrowed slightly (third trimester UW weight 13% lower than NW; OW 17.6% greater than NW). OW women reported 20.5% greater incomes compared to NW and 16.5% greater than UW; however, variation around the mean was also higher for overweight women’s incomes. Pregnancy glucose levels were similar across BMI groups, both by mean first trimester random glucose and by mean second trimester 50 g challenge test. Total pregnancy weight gain was qualitatively similar across groups, ranging from 10.5 kg (OW) to 11.5 (UW). No other differences were detected between the women by biological markers or sociodemographic factors.

**Figure 2:**
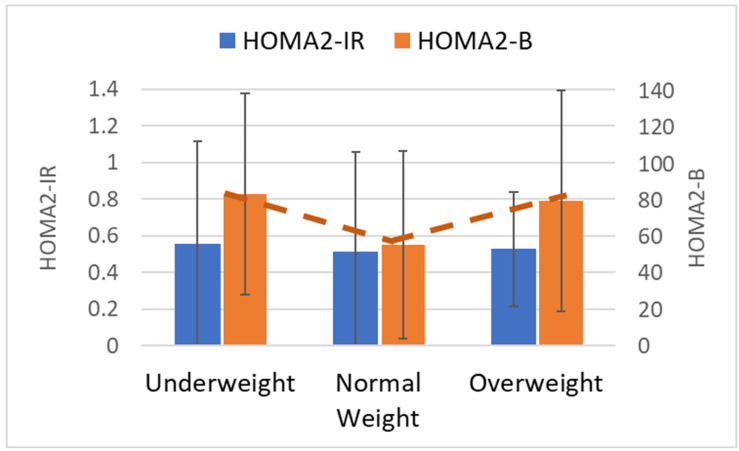
Assessment of static beta cell function and insulin resistance at the time of birth from cord blood. Static beta cell function (HOMA2-B, right-sided *y*-axis, orange bars) was lowest in normal weight and was associated with weight group in a U-shaped relationship, as illustrated by the dotted orange line. Insulin resistance (HOMA2-IR, shown on left *y*-axis, blue bars) did not vary between groups. Underweight *n* = 63; normal weight *n* = 43; overweight *n* = 29.

At birth, mean gestational age for the whole sample was 38.1 weeks (SD 1.7), similar across maternal BMI groups. Some anthropometric features were similar across groups including length and birth weight (Table 1). In contrast, cord blood glucose was over 15% greater in infants born to normal weight mothers (I_UW_ 84.9 mg/dL, SD 22.0; I_NW_ 100.5 mg/dL, SD 21.9 mg/dL; I_OW_ 88.5 mg/dL, SD 24.0). Cord HOMA2-B values were about 50% greater for infants born to underweight mothers (I_UW_ 83.5, SD 55.2) and 44% greater for those born to overweight mothers (I_OW_ 79.9, SD 60.8) compared to those born to normal weight mothers (I_NW_ 55.4, SD 51.5, Figure 2), forming a U-shaped relationship between maternal weight and HOMA2-B glucose levels. There were no differences in cord HOMA2-IR across BMI groups. 

**Figure 3:**
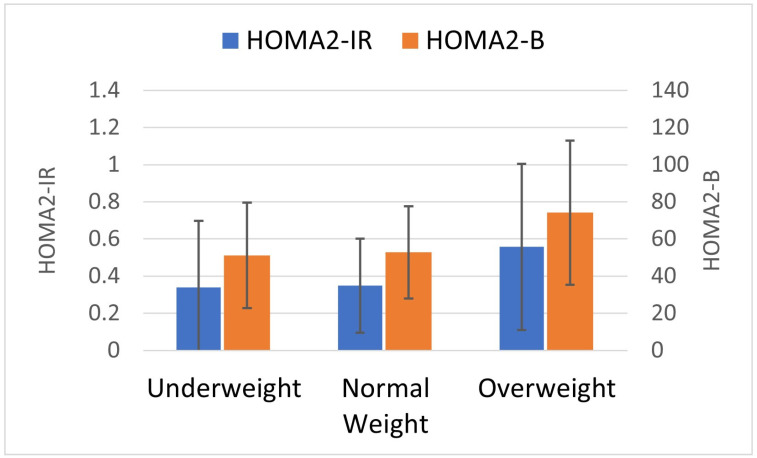
Infant static beta cell function and insulin resistance at 7 months postpartum. Static beta cell function (HOMA2-B, right-sided *y*-axis, orange bars) was lowest in underweight and normal weight. HOMA2-B was associated with weight group in a muted J-shaped relationship. Insulin resistance (HOMA2-IR, shown on left *y*-axis, blue bars) was higher in overweight compared to other groups. Underweight *n* = 63; normal weight *n* = 43; overweight *n* = 29.

Infant follow-up time was similar across maternal BMI groups, as reflected by similar mean infant age at the time of the second study visit (Table 2). Mean weight for age Z-score and length for age Z-score were similar across infants at the 7-month visit. The three groups of infants had similar fasting glucose levels but differing fasting insulin levels. Mean fasting insulin and mean fasting C-peptide were qualitatively higher in I_OW_ (C-peptide I_OW_ mean 259.1 pmol/L, SD 208.9 vs. I_NW_ 161.2 pmol/L, SD 114.9 vs. I_UW_ I_OW_ 157.3 pmol/L, SD 160.4). Unlike the other groups, HOMA2-IR levels were sustained in I_OW_ (HOMA2-IR at 7 months mean 0.56, SD 0.45; at birth 0.53, SD 0.31). I_OW_ had 59% greater mean HOMA2-IR and 41% greater HOMA2-B (74.2, SD 38.8) compared to the other groups (Figure 3, HOMA2-IR I_NW_ (0.35, SD 0.25) or I_UW_ (0.34, SD 0.36); HOMA2-B I_NW_ 52.8 SD 24.9 or I_UW_ 51.1, SD 28.4).

**Figure 4:**
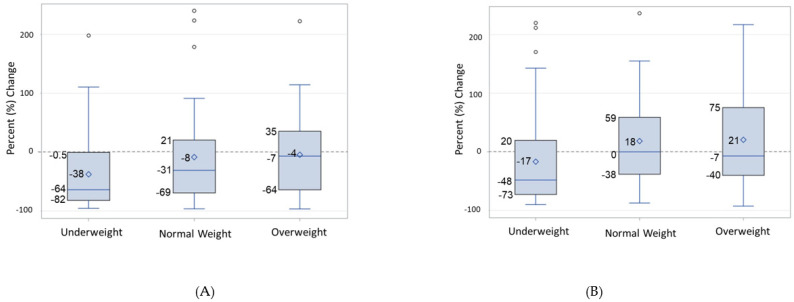
Median percent change in infant beta cell function and infant insulin resistance in first seven months after birth. (**A**) Percent change in HOMA2-IR. (**B**) Percent change in HOMA2-B. From birth to 7 months of age, static beta cell function decreased most in infants from underweight mothers compared to other infants, with parallel decreases in insulin resistance. The number of outliers for percent change that were omitted from this analysis (% change ≥ 250%) were underweight 4, normal weight 6, overweight 2. The number of outliers omitted from HOMA2-B percent change analysis (% change ≥ 250%) were underweight 0, normal weight 1, overweight 0.

Unlike the static measures of beta cell function at 7 months, the dynamic measure of beta cell function (DI) was greatest in infants from underweight mothers (I_UW_). To examine dynamic beta cell function, we first examined glucose tolerance test values at 30 min of the test. Mean 30 min glucose, insulin, and C-peptide were similar across weight groups. The mean insulinogenic index was greater in I_UW_ and in I_OW_ (15% and 27%, respectively) compared to I_NW_. Mean insulinogenic index was I_UW_ 0.17 nmol/mmol, SD 2.39 vs. I_NW_ 0.14 nmol/mmol, SD 2.20 vs. I_OW_ (0.18 nmol/mmol, SD 1.31, Table 2). Results were similar for the insulinogenic index calculated using insulin rather than C-peptide. The geometric mean oral disposition index was greatest for I_UW_ (mean I_UW_ 1.9 L/mmol, SD 2.5; I_NW_ 1.3 L/mmol, SD 2.6; I_OW_ 1.2 L/mmol, SD 3.7). Using the longitudinal measurements of HOMA2-IR and HOMA2-B, we assessed the within-group change in static beta cell function and insulin resistance during the first 7 months of life (Figure 4). The percent change per person in HOMA2-IR was greatest for I_UW_ median percent change: I_UW_ −64%; I_NW_ −31%; I_OW_ −7%). HOMA2-B decreased in I_UW_ (median: −48%; I_NW_ 0%; and I_OW_ -7%). I_OW_ and I_NW_ most closely maintained birth levels of HOMA2-B and HOMA2-IR over time, but not I_UW_. 

Multivariate linear regression supports the findings above for analyses at birth and at infants aged 7 months (Table 3). Maternal weight group was associated with HOMA2-B at birth (beta estimate 0.27, *p* = 0.007) but not HOMA2-IR at birth (beta estimate 0.15, *p* = 0.14). Adjustment by maternal education and for second trimester glucose challenge test glucose level improved the model R-square. Maternal pregnancy weight gain was not associated with birth HOMA2-B or HOMA2-IR. When infants were 7 months old, maternal weight group was not associated with the insulinogenic index or the disposition index. Crude maternal weight group was associated with HOMA2-B (beta estimate 0.14, *p* = 0.0048), but not maternal weight adjusted for other factors. Maternal weight group was not associated with HOMA2-IR at 7 months (beta estimate = 0.20, *p* = 0.11).

## 4. Discussion

This study provides evidence that maternal BMI may be associated with infant beta cell function at birth and at 7 months postpartum. At birth, static beta cell function, measured in cord blood, was associated with maternal BMI in a U-shaped pattern; infants from underweight and overweight mothers had higher beta cell function (possibly compensatory) compared to those from normal weight mothers, even while insulin resistance levels were similar across groups. At 7 months postpartum, we continued to observe an association of maternal weight to infants’ beta cell function. These associations qualitatively differed for static beta cell function (i.e., HOMA2-B at fasting) as compared to dynamic beta cell function (i.e., DI, in response to a glucose challenge). First, infant static beta cell function at 7 months was not associated with infant beta cell function in a U shape, but rather, more of a J shape reflecting higher HOMA2-B in overweight maternal BMI only. From the time of birth to 7 months postpartum, static beta cell function decreased most for the infants born to underweight mothers compared to other infants, decreases which occurred in parallel to decreases in insulin resistance. In contrast, infant disposition index at 7 months was greatest in infants born to underweight mothers compared to those born to normal weight or compared to overweight mothers (L-shaped association). Since I_UW_ had similar 7-month HOMA2-IR levels compared to I_NW_, one possible explanation for I_UW_ having the highest DI levels might be that infants born to underweight mothers produce more fasting insulin for metabolic needs as compared to I_NG_. 

Other studies support our findings that beta cell function in the next generation is related to maternal nutritional environment. The Pune Maternal Nutrition Study (PMNS) recruited 797 women who became pregnant and whose children received an oral glucose tolerance test at 6 and 18 years of age. Offspring glucose intolerance at 18 years (which developed in 30%) was associated with maternal weight (i.e., ‘ever underweight’), and young adults from mothers who reported ever being overweight had a lower prevalence of glucose intolerance [32]. Our findings that child beta cell function was associated with maternal weight could reflect the timing of pancreatic beta cell development. Beta cell replication is greatest during the first 2 years of life [33]; hence, our functional tests were administered while beta cell mass was still increasing. If maternal weight is associated with pancreatic beta cell function, it is possible that assessing children after an additional year or two of follow-up, i.e., after the most rapid period of beta cell replication, might more comprehensively reflect the impacts of maternal weight on pancreatic endocrine function. In the Pune Children’s Study, glucose intolerance at the age of 21 years was linked to differences in dynamic beta cell function years prior (i.e., at the age of 8 years) [32]. Those who later maintained normal glycemia experienced increased beta cell secretion, whereas there was little increase in such glucose-stimulated insulin secretion for those who became glucose intolerant. 

The maternal metabolic environment influences fetal growth and development through several pathways that include maternal glucose production and nutrient availability to the developing fetus [13,34,35]. Intrauterine undernutrition can disrupt the development of fetal organ structure and function, including organs responsible for insulin secretion and insulin sensitivity, prompting fetal adaptation favoring metabolically thrifty pathways to survive (i.e., thrifty phenotype) [36]. Metabolism in pregnant women may be a reflection of this adaptation; within the first two trimesters of pregnancy, low-BMI women from India may produce glucose 20% faster per kg compared to normal-BMI women from India, through pathways of glycogenolysis rather than gluconeogenesis [37]. Protein turnover also varies in pregnant women by weight class; overweight women may undergo greater protein turnover compared to those with lower BMI [38]. Overweight women may also have adipose tissue that secretes adipokines which have the potential to induce metabolic programming through several pathways, including fetal exposure to circulating adipokines. For example, leptin controls the development of hypothalamic neurocircuits and can lead to permanent changes in diet and exercise preferences [35]. Adiponectin can regulate fetal exposure to nutrients and, as a result, to fetal growth as well [13]. The various domains of the maternal exposome (e.g., both external and internal domains) could produce conditions such as epigenetic modifications, altered placental function, varied gut microbiome composition, or heightened metabolic inflammation, conditions that could have downstream effects on infant growth and development [39]. The extent to which the maternal metabolic environment influences pancreatic development will require further study.

Future studies should examine pathways of pancreatic development to identify targets of diabetes prevention, including beta cell replication (i.e., expansion to support metabolic demand), beta cell integrity (i.e., cell structure and machinery), and cell–cell communications that enhance insulin secretion. Mechanisms leading to suboptimal beta cell function include reduced pancreatic progenitor cells and/or epigenetic changes that lead to permanent alternations in beta cell function [8]. Future studies should examine the epigenetic signatures of a variety of tissues (such as adipose, brain, pancreas, and liver) that may heighten the risk of metabolic diseases. 

This study has several strengths. First, this study had an excellent retention rate of 92%. Second, our selected measures of beta cell function, including the oral disposition index, are rigorous and predictive of future diabetes in adolescents and adults [30]. Third, this study is among the first to implement a novel protocol for a standardized OGTT in a very young age group (infants less than one year old) for rigorous assessment of beta cell function. Two other studies have reported using glucose or meal tolerance testing to assess endocrine function in infants. Soto et al. [40] assessed infants for the exposure of small for gestational age compared to normal birth size. They followed infants and, at one year of age, provided a short intravenous glucose tolerance test after an overnight fast, with 25% dextrose solution administered at 0.5 g/kg by continuous infusion over 3 min and blood sampled during the first 10 min of the test for glucose and insulin. In a second study, healthy term infants were given meal tolerance tests between 4 and 8 months of age in a diet trial of follow-on formula [41]. Infants fasted for at least three hours before the test meal, and blood samples at 60 min after the start of formula feeding were examined for insulinemia. 

A variety of study limitations exists, including the possible misclassification of mothers’ weight groups due to the use of first trimester weight, which is the weight taken after the pregnancy began. Prepregnancy weights were unavailable; however, representative data from India suggest that Indian women on average do not gain appreciable weight in their first trimester, and the distribution of prepregnancy weight is very close to the empirical distribution for women’s weight at three months gestation [42]. Second, while this analysis included an overweight group, the sample did not include an adequate sample of obese women (n = 2), and thus, interpretations are limited to overweight, non-obese BMI. Another limitation of the study was that follow-up ended during the first year of infant age, yet pancreatic endocrine mass develops most across the first two years of life, and, therefore, observations in this study may be too early to quantify the association of maternal weight on infant pancreatic function. Other limitations include lack of tools to measure pancreatic endocrine cell mass, prepregnancy diet, physical activity, or other indicators of nutritional status. 

The findings of this study have clinical significance. Approximately 42% of prepregnant women in India are underweight and commonly complete pregnancy weighing even less than women beginning pregnancy in other countries [42]. Moreover, the unique heterogeneity of diabetes phenotypes suggest that Asian Indians are more commonly comprised of severe insulin-deficient phenotypes than other populations [43]. As such, Asian Indian women are more likely to be underweight and have a metabolic phenotype of pancreatic dysfunction. The timing of this study, within the first year of postnatal life, suggests that maternal weight groups have an impact on infant basal metabolism. The results of this study, taken with other studies, also suggest that challenges with compensatory insulin secretion could be visible later in childhood among individuals whose mothers are underweight. On the other end of the BMI spectrum, more women are becoming overweight in India [44], and evidence here provides the possible impacts of pregnancy overweight on infants’ sustained elevations of infant insulin levels. While questions remain regarding the time-course of healthy vs. maladaptive pancreatic development, existing evidence supports ongoing antenatal care strategies to improve nutritional status in pregnancy.

In conclusion, this study provides evidence of specific early-life endocrine features associated with the maternal nutritional environment. Compared to infants born to normal weight mothers, infants from underweight mothers and overweight mothers exhibit similar patterns of beta cell function at birth. During the first 7 months of postnatal life, changes to metabolic measures were most dramatic for infants born to underweight mothers. After an oral glucose tolerance test, infants born to underweight mothers had the highest levels of dynamic beta cell function compared to infants born to normal weight or overweight mothers—suggesting a compensatory response. Future research should assess the long-term implications of heightened insulin secretion in infancy compared to infants born to normal weight mothers. The totality of findings here warrants further examination of intergenerational risk of pancreatic poor beta cell structure and function.

## Figures and Tables

**Table 1:** Baseline characteristics of mother–infant dyads.

Characteristics	Underweight Mothers (n = 63)		Normal Weight Mothers (n = 43)		Overweight Mothers (n = 29)		% Difference Underweight vs. Normal Weight	% Difference Overweight vs. Normal Weight
**M** **other**	Mean (SD) or *n* (%)		Mean (SD) or *n* (%)		Mean (SD) or *n* (%)			
Age at delivery (yrs)	23.0	3.0	22.7	3.0	23.8	3.0	1.3	4.8
Height (cm)	153.1	5.7	151.8	5.5	152.0	5.7	1.3	0.2
Family history of diabetes (n, %)	14	22.2	11	25.6	10	34.5	−3.4	8.9
Highest education: 10th grade or less (n, %)	45	71.4	3.0	58.1	13	44.8	13.3	−13.3
Income (Indian Rupee)	10,920	4330	10,560	3100	12,720	9680	3.4	20.5
Religion: Hindu (n, %)	60	95.2	36	83.7	25	86.2	11.5	2.5
Random Glucose–1st trimester (mg/dL)	88.0	14.2	88.7	17.2	87.7	11.9	−0.8	−1.1
Glucose from 50 g Glucose Challenge Test (mg/dL)	103.6	23.2	101.3	23.9	106.2	23.8	2.3	4.8
BMI 1st trimester (kg/m^2^)	17.0	1.2	20.6	1.3	25.1	1.3	−17.5	21.8
Weight 1st trimester (kg)	39.7	4.2	47.5	4.4	58.2	6.7	−16.4	22.5
Weight 2nd trimester (kg)	42.1	4.6	49.0	4.9	59.3	6.7	−14.1	21.0
Weight 3rd trimester (kg)	50.3	5.7	57.8	6.3	68.0	7.8	−13.0	17.6
Total Weight Gain (kg)	11.5	3.9	11.1	3.5	10.5	4.2	3.6	−5.4
**Infant**								
Gestational Age at birth (weeks)	38.2	1.7	38.0	1.9	37.9	1.4	0.5	−0.3
Sex: female (n, %)	31	49.2	24	55.8	14	48.3	−6.6	−7.5
Preterm birth (n, %)	7	11.1	9	20.9	7	24.1	−9.8	3.2
Birth weight (g)	2680	380	2820	410	2930	430	−5.2	3.7
Weight for Age Z score	−1.3	0.8	−1.2	1.1	−0.9	1.0	−0.1 *	0.3 *
Length at birth (cm)	47.0	2.3	47.2	2.7	46.7	3.6	−0.4	−1.1
Length for Age Z score	−1.3	1.2	−1.3	1.4	−1.3	1.3	0 *	0 *
Head Circumference (cm)	33.4	2.5	33.6	3.2	34.6	3.5	−0.6	3.0
Head Circumference for Age Z score	−0.6	2.1	−0.4	2.7	0.3	3.0	−0.2 *	0.7 *
Glucose at birth (mg/dL)	84.9	22.0	100.5	21.9	88.5	24.0	−15.5	−11.9
Insulin at birth (mIU/L)	5.4	8.8	4.9	7.1	4.8	5.7	10.2	−3.0
C-peptide at birth (pmol/L)	260.3	243.5	231.0	245.0	246.3	153.6	12.7	6.6
HOMA2-IR at birth (mean, SD)	0.56	0.56	0.51	0.55	0.53	0.31	9.2	3.1
HOMA2-B at birth (mean, SD)	83.5	55.2	55.4	51.5	79.9	60.8	50.8	44.2

All HOMA2 indices shown were calculated using C-peptide. * For Z-score variables, differences in Z scores are shown.

**Table 2:** Infant characteristics at 7 months of age.

Infant Characteristics	Underweight Mothers (n = 63)		Normal Weight Mothers (n = 43)		Overweight Mothers (n = 29)		% Difference Underweight vs. Normal Weight	% Difference Overweight vs. Normal Weight
	Mean (SD) or *n* (%)		Mean (SD) or *n* (%)		Mean (SD) or *n* (%)			
Age, months ~	7.2	0.6	7.5	1.1	7.5	1.0	−4.0	0.7
Weight (kg)	7.1	0.9	7.4	0.9	7.4	1.0	−3.3	1.2
Weight for Age (Z-score)	−1.1	1.1	−0.9	1.1	−0.8	1.1	0.0 *	0.0 *
Length (cm)	66.7	2.5	67.1	2.3	67.6	2.6	−0.7	0.7
Length for Age (Z-score)	−0.8	1.1	−0.7	1.0	−0.6	1.0	0.0 *	0.0 *
Weight for Length (Z-score)	−0.8	1.1	−0.5	1.2	−0.6	1.2	0.0 *	0.0 *
Breast Feeding: yes	50.0	79.4	37.0	86.1	24.0	82.8	−6.7	−3.3
Glucose 0 min (mg/dL)	79.7	9.5	82.5	12.2	82.7	15.5	−3.4	0.2
Glucose 30 min (mg/dL)	105.7	22.0	114.0	21.7	109.8	22.6	−7.3	−3.7
Insulin 0 min (mIU/L)	2.6	3.5	2.2	2.4	4.5	7.9	15.4	103.6
Insulin 30 min (mIU/L)	8.9	7.1	9.7	8.1	10.2	7.3	−8.5	4.8
C-Peptide 0 min (pmol/L)	157.3	160.4	161.2	114.9	259.1	208.9	−2.4	60.7
C-Peptide 30 min (pmol/L)	395.3	231.7	443.8	280.4	471.4	251.0	−10.9	6.2
HOMA2-IR	0.34	0.36	0.35	0.25	0.56	0.45	−3.1	59.1
HOMA2-B	51.1	28.4	52.8	24.9	74.2	38.8	−3.1	40.6
Insulinogenic Index (using C-peptide, nmol/mmol)	0.17	2.39	0.14	2.20	0.18	1.31	15.0	27.1
Disposition Index (using C-peptide, L/mmol)	1.9	2.5	1.3	2.6	1.2	3.7	49.2	−6.8

Geometric means and SDs reported for disposition index. (%). ~ Month defined as 30 days. * For Z-score variables, differences in Z scores are shown.

**Table 3:** Associations of maternal weight group and insulin resistance or beta cell function.

	Beta Maternal Weight	SE Maternal Weight	P(t) Maternal Weight	R-Squared	Model p (F)
**Models of Static Indices at Birth**					
Model Set 1: Outcome HOMA2-IR					
Model 1a: Birth HOMA2-IR ~ Maternal Weight Group	0.15	0.10	0.14	0.02	0.14
Model 1b: Birth HOMA2-IR ~ Maternal Weight Group + Pregnancy Weight Gain	0.15	0.10	0.14	0.02	0.32
Model 1c: Birth HOMA2-IR ~ Maternal Weight Group + Education	0.16	0.10	0.10	0.04	0.08
Model 1d: Birth HOMA2-IR ~ Maternal Weight Group + 2nd trimester GCT glucose	0.20	0.10	0.05	0.03	0.15
,Model 1e: Birth HOMA2-IR ~ Maternal Weight Group + Birth HOMA2-B	−0.05	0.07	0.49	0.53	<0.0001
Model Set 2: Outcome HOMA2-B					
Model 2a: Birth HOMA2-B ~ Maternal Weight Group	0.27	0.10	0.007	0.05	0.007
Model 2b: Birth HOMA2-B ~ Maternal Weight Group + Pregnancy Weight Gain	0.28	0.10	0.007	0.06	0.02
Model 2c: Birth HOMA2-B ~ Maternal Weight Group + Education	0.29	0.10	0.004	0.07	0.01
Model 2d: Birth HOMA2-B ~ Maternal Weight Group + 2nd trimester GCT glucose	0.35	0.10	0.0005	0.1	0.002
Model 2e: Birth HOMA2-B ~ Maternal Weight Group + Birth HOMA2-IR	0.17	0.07	0.02	0.55	<0.0001
**Models of Static Indices at 7 months**					
Model Set 3: Outcome HOMA2-IR					
Model 3a: HOMA2-IR ~ Maternal Weight Group	0.20	0.13	0.11	0.02	0.11
Model 3b: HOMA2-IR ~ Maternal Weight Group + Pregnancy Weight Gain	0.20	0.13	0.12	0.03	0.18
Model 3c: HOMA2-IR ~ Maternal Weight Group + Education	0.16	0.13	0.20	0.02	0.20
Model 3d: HOMA2-IR ~ Maternal Weight Group + 2nd trimester GCT glucose	0.22	0.13	0.09	0.03	0.17
Model 3e: HOMA2-IR~ Maternal Weight Group +HOMA2-B	−0.02	0.06	0.66	0.82	<0.0001
Model 3f: HOMA2-IR~ Maternal Weight Group +Birth HOMA2-B	0.25	0.13	0.05	0.03	0.11
Model Set 4: Outcome HOMA2-B					
Model 4a: HOMA2-B~ Maternal Weight Group	0.14	0.07	0.05	0.03	0.048
Model 4b: HOMA2-B~ Maternal Weight Group + Pregnancy Weight Gain	0.14	0.07	0.05	0.03	0.10
Model 4c: HOMA2-B~ Maternal Weight Group + Education	0.12	0.07	0.10	0.03	0.16
Model 4d: HOMA2-B~ Maternal Weight Group + GCT glucose	0.14	0.07	0.06	0.03	0.15
Model 4e: HOMA2-B~ Maternal Weight Group + HOMA2-IR	0.04	0.03	0.21	0.82	<0.0001
Model 4f: HOMA2-B ~ Maternal Weight Group + Birth HOMA2-IR	0.15	0.07	0.04	0.03	0.11
**Models of Dynamic Indices at 7 Months**					
Model 5a: Insulinogenic Index~ Maternal Weight Group	0.12	0.12	0.31	0.009	0.31
Model 5b: Insulinogenic Index~ Maternal Weight Group + HOMA2-IR	0.09	0.11	0.44	0.17	<0.0001
Model 5c: Disposition Index ~ Maternal Weight Group	0.02	0.13	0.87	0.0002	0.87
Model 5d: Disposition Index ~ Maternal Weight Group + HOMA2-IR	0.08	0.11	0.48	0.31	<0.0001

Legend Table 3. Log-transformed values for HOMA2-IR, HOMA2-B, insulinogenic, and disposition indices were used in all models. Maternal weight group was modeled as a categorical variable of 3 levels (2 df). Abbreviations: GCT, glucose challenge test.

## Data Availability

The data presented in this study are available on request from the corresponding author. The data are not publicly available due to maintain privacy of the participants.
